# The diagnostic value of staging laparoscopy in gallbladder cancer: a nationwide cohort study

**DOI:** 10.1186/s12957-022-02880-z

**Published:** 2023-01-14

**Authors:** Mike van Dooren, Elise A. J. de Savornin Lohman, Eva Brekelmans, Pauline A. J. Vissers, Joris I. Erdmann, Andries E. Braat, Jeroen Hagendoorn, Freek Daams, Ronald M. van Dam, Marieke T. de Boer, Peter B. van den Boezem, Bas Groot Koerkamp, Philip R. de Reuver

**Affiliations:** 1 Department of Surgery, Radboud university medical center, P.O. Box 9101, Internal Code 618, 6500 HB Nijmegen, the Netherlands; 2 Department of Research, Netherlands Comprehensive Cancer Organization (IKNL), P.O. Box 19079, 3501 DB Utrecht, the Netherlands; 3 Department of Surgery, Amsterdam University Medical Centers, P.O. Box 22660, 1100 DD Amsterdam, the Netherlands; 4 Department of Surgery, Leiden University Medical Center, P.O. Box 9600, 2300 RC Leiden, the Netherlands; 5 Department of Surgery, UMC Utrecht Cancer Center, P.O. Box 85500, 3508 GA Utrecht, the Netherlands; 6 Department of Surgery, St. Antonius Hospital, P.O. Box 2500, 3430 EM Nieuwegein, the Netherlands; 7 Department of Surgery, Maastricht University Medical Center +, P.O. Box 5800, 6202 AZ Maastricht, the Netherlands; 8 Department of Hepatobiliary and Transplant Surgery, University Medical Center Groningen, P.O. Box 30.001, 9700 RB Groningen, the Netherlands; 9 Department of Surgery, Erasmus MC, P.O. Box 2060, 3000 CB Rotterdam, the Netherlands

**Keywords:** Gallbladder neoplasms, Gallbladder carcinoma, Staging laparoscopy

## Abstract

**Background:**

Disseminated disease (DD) is often found at (re-)exploration in gallbladder cancer (GBC) patients. We aimed to assess the yield of staging laparoscopy (SL) and identify predictors for DD.

**Methods:**

This retrospective study included patients from all Dutch academic centres with primary GBC (pGBC) and incidentally diagnosed GBC (iGBC) planned for (re-)resection. The yield of SL was determined. In iGBC, predictive factors for DD were assessed.

**Results:**

In total, 290 patients were included. Of 183 included pGBC patients, 143 underwent laparotomy without SL, and 42 (29%) showed DD perioperatively. SL, conducted in 40 patients, identified DD in eight. DD was found in nine of 32 patients who underwent laparotomy after SL.

Of 107 included iGBC patients, 100 underwent laparotomy without SL, and 19 showed DD perioperatively. SL, conducted in seven patients, identified DD in one. Cholecystitis (*OR* = 4.25; 95% *CI* 1.51–11.91) and primary R1/R2 resection (*OR* = 3.94; 95% *CI* 1.39–11.19) were independent predictive factors for DD.

**Conclusions:**

In pGBC patients, SL may identify DD in up to 20% of patients and should be part of standard management. In iGBC patients, SL is indicated after primary resection for cholecystitis and after initial R1/R2 resection due to the association of these factors with DD.

## Introduction

Although gallbladder cancer (GBC) is relatively rare in Western populations, it is the most common biliary tract malignancy. Its overall 5-year survival rate is less than 5%, but the prognosis is very dependent of the disease stage [1]. Symptoms are often non-specific and do not occur until late in the disease course. Patients are frequently diagnosed at a stage in which extended resection is necessary to achieve a radical resection.

Up to 70% of patients with GBC is diagnosed incidentally (iGBC), during or after cholecystectomy for a presumed benign indication, such as cholecystolithiasis or cholecystitis [2, 3]. Due to the risk of residual disease, re-resection is recommended in patients with pathologically confirmed stages T1b, T2, and T3 disease to improve survival [4]. The remaining 30% are diagnosed primarily with GBC (pGBC), mainly through radiological imaging.

In patients with disseminated disease (DD, defined as either locally unresectable or metastatic disease), resection does not improve survival [5]. To detect DD and improve selection of patients for surgery, imaging techniques such as computed tomography (CT) and magnetic resonance imaging (MRI) are used [6]. Unfortunately, the sensitivity of imaging for DD in GBC patients is low, with reported sensitivities between 70 and 80% [7, 8]. Therefore, DD is frequently detected during exploratory laparotomy [9].

Routine staging laparoscopy (SL) may avoid an unnecessary laparotomy. Although some studies report on the use of SL in GBC, the majority of previous studies stem from single-centre series, and generalizability may be limited [10–14]. Moreover, only one study investigated the value of SL and potential predictive factors for DD in iGBC [12].

This study aims to assess the value of SL for both pGBC and iGBC to define its role in the current treatment strategy for GBC patients. In addition, this study aims to identify predictors for DD in iGBC patients.

## Methods

### Patient inclusion

In this retrospective database study, patients from all seven academic centres in the Netherlands diagnosed with GBC between November 1999 and May 2018 were eligible for inclusion. Eligible patients were identified through the Netherlands Cancer Registry (NCR), which contains data on all newly diagnosed malignancies, by creating a list of all patients with a GBC diagnosis between November 1999 and May 2018 per academic centre. To make groups of patients with potentially resectable pGBC and patients with iGBC planned for re-resection, hospital medical records of all eligible patients were examined. pGBC patients deemed unresectable due to DD on radiological imaging as well as iGBC patients not planned for re-resection were excluded from the study. These data include a patient identification number, treatment hospital, and date of diagnosis. The NCR is maintained by the Netherlands Comprehensive Cancer Organization (IKNL) and is notified of newly diagnosed patients by the automated pathological archive (PALGA) [15], the nationwide network and registry of histo- and cytopathology of the Netherlands, and supplemented by data from the National Archive of Hospital Discharge Diagnosis. Of eligible patients identified from the NCR, patient information was collected from hospital medical records. Two groups of patients were included; the first group contained patients with pGBC planned for resection with curative intent. pGBC was defined as GBC identified at radiological imaging, including US (ultrasound), CT, FDG PET-CT (fluorodeoxyglucose PET-computed tomography), and MRI. The second group contained patients with iGBC planned for re-resection. iGBC was defined as unsuspected gallbladder cancer found during cholecystectomy for benign gallbladder disease or postoperative histopathological analysis. Re-resection was defined as additional GBC-directed surgery with curative intent within 6 months after primary surgical exploration. The study was approved by an accredited Medical Research Ethics Committee (METC Oost-Nederland) with number 2017-3912.

### Diagnostic and treatment procedures

In the Netherlands, diagnostic and treatment procedures are described in hospital-specific guidelines, which in turn are based on guidelines published by the Dutch National Workgroup for Gastrointestinal Tumours [16]. The first version of this national guideline was published in 2004 and a revised version was published in 2013. Recommended and performed diagnostic and treatment procedures may therefore have varied per patient and per hospital. SL was defined as a laparoscopic operation in which resectability of the gallbladder bed is assessed and in which lymph nodes, liver, peritoneum, omentum and other visible abdominal organs are examined for signs of metastatic disease. A frozen section procedure was performed of masses suspected of metastasis. Laparoscopic ultrasonography of the liver was performed in some but not all cases. Both the one-stage approach, with direct continuation to laparotomy, and two-stage approach, with scheduling of laparotomy on a future date, were described. In iGBC patients, SL was performed after initial cholecystectomy and before (possible) re-resection.

### Data collection and variable definitions

Patient characteristics such as age, gender, ASA Physical Status Classification [17], symptoms, previous gallbladder disease, organ invasion and T- and N-stage on preoperative imaging, description and results of operative procedures, tumour characteristics, and other histopathological findings and follow-up data were extracted from patient medical records. The number of days between SL and (re-)laparatomy was recorded.

### Outcomes

The primary outcome of this study was the yield of SL, defined as the proportion of patients with the presence of DD during SL. The yield was assessed separately for pGBC and iGBC patients, as diagnostic and treatment pathway and expected yield for these groups are different.

Secondary outcomes were also determined separately for pGBC and iGBC patients. In pGBC patients, the location of DD and the resection rate was determined. Resection rate was defined as the proportion of patients that underwent laparotomy for resection with curative intent with a macroscopic radical resection. In the pGBC group, patient characteristics were compared between patients who did and did not undergo SL. For iGBC, predictive factors for DD were analysed in order to investigate which group of iGBC patients would benefit most from SL.

### Statistical analysis

Baseline characteristics were described using frequencies and percentages for categorical variables, and median value and range for continuous variables. To compare SL- and no SL-groups, Chi-squared tests were used for categorical variables and the Student’s t-test was used for continuous variables. Univariable and multivariable analysis were used to identify individually predictive factors for presence of DD during laparotomy. All clinical or histopathologic findings with a significance level of P≤0.1 in univariable analysis were included into a multivariable model using backwards Wald logistic regression. Results from the regression analysis were reported as Odds ratios (ORs) with 95% confidence interval (CI). All analyses were performed with SPSS version 26.0 [18]. *P*-values ≤ 0.05 were considered statistically significant.

## Results

**Fig. 1:**
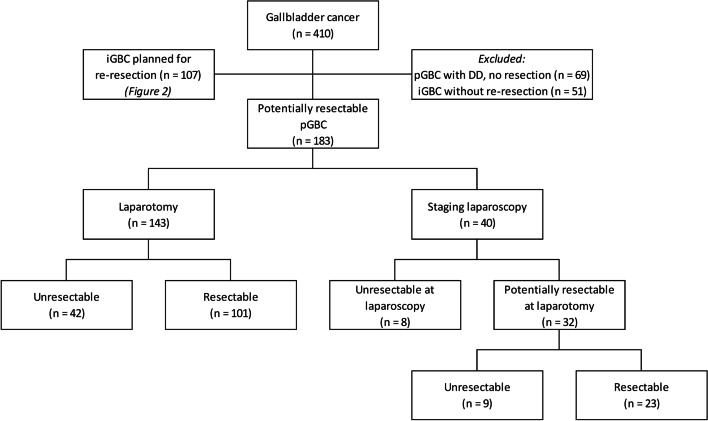
Patients with primary GBC

A total of 410 patients were identified through the NCR. Of those, 183 patients were diagnosed with potentially resectable pGBC, and 107 patients were diagnosed with iGBC and were planned for re-resection. A total of 120 patients were excluded; 69 patients were deemed unresectable due to DD on radiological imaging, and 51 patients with iGBC were not planned for re-resection (Fig. 1).

### Primary GBC

Of the 183 included patients with pGBC, 143 (78%) underwent laparotomy without SL. Resection was performed in 101 of these 143 patients (71%). In 42 patients (29%), resection was not performed due to DD, a combination of liver metastases (*n* = 11), peritoneal seeding (*n* = 10), lymph node metastases (*n* = 10), and local unresectability (*n* = 18) (Fig. 1).

Liver invasion on preoperative imaging was present more often in the SL group (20/40 vs 45/143, *p* = 0.030), as well as invasion into other organs (18/40 vs 34/143, *p* = 0.008) (Table 1).

**Table 1:** Baseline characteristics of patients with primary GBC

	Staging laparoscopy *N* = 40	No laparoscopy *N* = 143	*p*-value
Baseline characteristics^a^			
Age, years, median (range)	64 (42–86)	67 (33–89)	0.088
Gender			
Female	16 (40.0%)	93 (65.0%)	**0.004**
Male	24 (60.0%)	50 (34.7%)	
Symptoms			
Jaundice	17 (42.5%)	52 (36.4%)	0.479
Abdominal pain	29 (72.5%)	76 (53.1%)	**0.029**
Weight loss	11 (27.5%)	34 (23.8%)	0.629
ASA-classification			0.576
1	6 (15.0%)	30 (21.0%)	
2	25 (62.5%)	72 (50.3%)	
3	8 (20.0%)	36 (25.2%), 2	
4	1 (2.5%)	(1.4%)	
Primary sclerosing cholangitis	2 (5.0%)	7 (4.9%)	0.978
Characteristics on imaging^b^			
Cholecystitis	0 (0.0%)	10 (7.0%)	0.085
Cholecystolithiasis	8 (20.0%)	50 (35.0%)	0.072
Gallbladder polyp	1 (2.5%)	6 (4.2%)	0.621
N1/2 stage	9 (22.5%)	32 (22.4%)	0.456
Liver invasion	20 (50.0%)	45 (31.5%)	**0.030**
Other organ invasion	18 (45.0%)	34 (23.8%)	**0.008**
cT stage			0.104
1–2	4 (10.0%)	24 (16.8%)	
3-4	18 (45.0%)	41 (28.7%)	
X	*18 (45.0%)*	*78 (54.5%)*	

^a^Baseline characteristics were defined as general patient demographics at diagnosis; median age in years at date of diagnosis, gender, symptoms at diagnosis, ASA physical status classification grade, and history of primary sclerosing cholangitis. ^b^Characteristics as described in preoperative imaging reports were cholecystitis, cholecystolithiasis, the presence of gallbladder polyps, pathological regional or distant lymph nodes, and invasion of the liver or other organs by the tumour and T stage. When unknown or not derivable from imaging, T stage was defined as Tx

Forty of the 183 included pGBC patients (22%) underwent SL and DD was detected in eight (20%, 8/40). Reasons for DD were the presence of (a combination of) liver metastases (*n* = 3), peritoneal seeding (*n* = 3), distant lymph node metastases (*n* = 1), and local unresectability (*n* = 5). Thirty-two patients were considered resectable after SL and subsequently underwent laparotomy. DD was detected during laparotomy in nine out of 32 patients; due to liver metastases (*n* =2), peritoneal seeding (*n* = 1), lymph node metastases (*n* = 2), and local unresectability (*n* = 4) (Fig. 1).

The resection rate after SL did not differ significantly from the resection rate in patients who underwent laparotomy without SL (23/32 vs. 101/143; *p* = 0.89). The number of days between SL and resection was not significantly different between patients who did and did not have DD upon laparotomy: 13 days (range 0–43) in patients with DD and 16 days (range 0–190) without DD (*p* = 0.52). In 16 patients, SL and resection were performed in one session.

### Incidental GBC

**Fig. 2:**
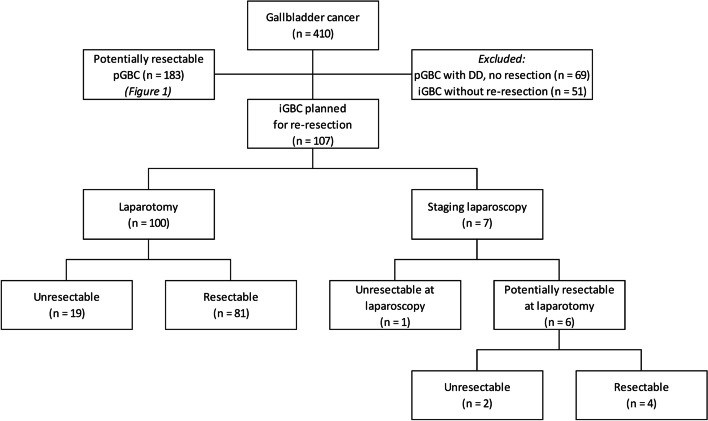
Patients with incidental GBC

Of the 107 included patients with iGBC, 100 (93%) underwent laparotomy without SL. Re-resection was performed in 81 of these 100 patients (81%). In 19 patients (19%), re-resection was not performed due to DD; peritoneal seeding (*n* = 11), lymph node metastases (*n* = 4), liver metastases (*n* = 1), a combination of lymph node and liver metastases (*n* = 1), and a combination of lymph node and peritoneal metastases (*n* = 2) (Fig. 2).

Seven of the 107 included iGBC patients (7%) underwent SL and yielded DD in one due to peritoneal metastases. Six patients were considered resectable by SL and subsequently underwent laparotomy. DD was detected during laparotomy in two out of six patients; peritoneal metastases in one, and a distant lymph node metastasis in another (Fig. 2). Overall, 22 of 107 iGBC patients had DD (21%). The median time between primary surgery and re-resection in iGBC patients was 75 days (range 0–297).

Factors predictive for the presence of DD in univariable analysis were cholecystitis, T3 disease, perineural invasion, positive lymph node status, and R1/R2 resection at primary exploration (Table 2). Multivariable logistic regression showed that cholecystitis (*OR* 4.25, 95% C*I* = 1.51–11.91; *p* = 0.006) and R1/R2 resection at primary surgery (*OR* 3.94, 95% *CI* = 1.39-11.19; *p* = 0.010) were independent predictive factors for DD at re-resection.

**Table 2:** Comparison between patients with disseminated disease and patients without disseminated disease after incidental GBC

Characteristics^a^	DD-group (*N* = 22)	Non-DD group (*N* =85)	*p*-value Univariable	*p*-value Multivariable	95% CI	OR
Age, years, median (range)	64 (49–81)	63 (36–81)	0.631			
Gender						
Female	19 (86.4%)	60 (70.6%)	0.134			
Male	3 (13.6%)	25 (29.4%)				
Symptoms						
Jaundice	2 (9.1%)	0 (0.0%)				
Abdominal pain	5 (22.7%)	18 (21.2%)	0.425			
Weight loss	0 (0.0%)	3 (3.5%)				
Cholecystitis	12 (54.5%)	18 (21.2%)	**0.002**	**0.006**	1.51–11.91	4.25
Cholecystolithiasis	8 (36.4%)	36 (42.4%)	0.582			
Primary sclerosing cholangitis	0 (0.0%)	4 (4.7%)	0.300			
Gallbladder polyp	0 (0.0%)	4 (4.7%)	0.300			
Type of surgery						
Laparoscopic cholecystectomy	16 (72.7%)	69 (81.2%)				
Open cholecystectomy	5 (22.7%)	15 (17.6%)	0.210			
Other	1 (4.5%)	1 (1.2%)				
Perioperative bile spill	5 (22.7%)	22 (25.9%)	0.761			
R1/R2 resection	15 (68.1%)	29 (34.1%)	**0.004**	**0.010**	1.39–11.19	3.94
Time between primary surgery and re-resection, days, median (range)	83 (5–132)	72 (0–164)	0.643			
pT stage						
1	1 (4.5%)	11 (12.9%)				
2	12 (54.5%)	62 (72.9%)	**0.015**	0.562	0.55–3.02	1.29
3	9 (40.9%)	12 (14.1%)				
pN stage						
0	2 (9.1%)	28 (32.9%)				
1	6 (27.2%)	14 (16.5%)	**0.074**	0.516	0.77–1.68	1.14
X	14 (63.6%)	43 (50.6%)				
Perineural invasion	10 (45.5%)	19 (22.4%)	**0.030**	0.110	0.83–6.30	2.29
Vascular invasion	8 (36.4%)	19 (22.4%)	0.178			

*Disease after incidental GBC*. ^a^Characteristics compared in univariable analysis were median age in years at date of diagnosis, gender, symptoms at diagnosis, cholecystitis or cholecystolithiasis on imaging, history of primary sclerosing cholangitis, gallbladder polyp on imaging, type of primary surgery, bile spill during or (macroscopic or microscopic) irradicality of primary surgery, median time in days between primary surgery and re-resection, pathological T and N stage after primary surgery, and perineural or vascular invasion as described in the pathology report. If no lymph nodes were resected, pN stage was defined as Nx

## Discussion

This study assessed the yield of SL before laparotomy in patients with pGBC and patients with iGBC. Of 183 patients with pGBC planned for resection, 143 (78%) did not undergo SL, 42 of which (29%) showed DD at laparotomy. Of 40 pGBC patients where SL was performed before planned resection, DD was found in eight.

Of 107 included iGBC patients, 100 (93%) did not undergo SL before planned re-resection, 19 of which (19%) showed DD at laparotomy. DD was found in one of seven iGBC patients that underwent SL before re-resection. This study identified cholecystitis and R1/R2 resection at primary cholecystectomy as independent predictive factors of DD in iGBC patients.

Regarding the use of SL in pGBC, results of previous publications are diffuse. The yield of SL in pGBC reported was generally higher than the present study and ranged from 23 to 62% [9–11, 13, 14]. A study from India including 409 pGBC patients undergoing SL reported a yield of 23.2%, which accords with our findings [10].

Studies assessing the yield of SL before re-resection in patients with iGBC are scarce. As iGBC is often diagnosed at an earlier stage, a lower yield for SL in comparison with pGBC is expected. A series of 136 iGBC patients treated in the Memorial Sloan-Kettering Cancer Center [12] found a yield of SL of only 4.3%. The authors reported that a higher T stage, positive resection margin, and poor tumour differentiation were found to be independent predictors of DD. In a prior paper from the same group, SL demonstrated DD in 2 out of 10 iGBC patients [14].

It must be noted that nine of 32 pGBC patients deemed potentially resectable during SL showed DD at laparotomy. This can be partially explained due to the difficulty of estimating resectability of locally advanced disease in SL, which was the reason for unresectability in four of the nine. No correlation between delayed timing of laparotomy after SL and the presence of DD at laparotomy was found in this study. In pGBC, the resection rate did not differ between patients with and without SL (72% vs.71%; *p* = 0.89). However, patients who did receive SL had signs of advanced disease (i.e. liver invasion) on preoperative imaging more frequently. Since SL is generally conducted in higher-stage patients, this may result in selection bias and explain the equal resectability rates.

In iGBC patients, yield of SL was lower; peritoneal metastases were detected by SL in one of seven patients. SL missed DD in two patients due to lymph node metastases. DD was most often present in patients with R1/R2 resection or cholecystitis. Possibly, the relation of cholecystitis with DD stems from its higher chance of bile spill during initial cholecystectomy, which has been described as a risk factor for DD [19].

Although previous studies comment on the usefulness of FDG PET-CT in the workup of patients with GBC, guidelines in the Netherlands only recommend its use when doubt regarding the presence of distant metastases exists on regular CT [20]. Also, there have been significant improvements in imaging technology during the inclusion period. These factors might have a positive effect on current radiological examination of disseminated disease and might therefore introduce bias and affect the yield of staging laparoscopy.

Based on the outcome of this study, we recommend SL for every patient with suspected pGBC in whom resection is considered as its yield is significant and the time investment is limited. Since the value of SL in iGBC seems moderate, SL should be considered in patients with high risk of DD (i.e. patients with cholecystitis or initial R1/R2 resection).

Due to the low incidence of GBC, international collaboration is of vital importance to obtain data on a larger cohort of patients. Currently, a large international study on the operative management of early gallbladder cancer, the OMEGA study, is being set up to further improve our knowledge on the treatment of gallbladder cancer.

Strengths of the present study include the nationwide design, which may provide outcomes which more accurately reflect the overall population compared to results from studies that only include data from high volume expert centres. Moreover, it is the first paper to research this topic in a European population and examines the use of SL in both pGBC and iGBC patients. There are some limitations to the current study. Primarily, the interpretation of results of a retrospective cohort study is vulnerable to selection bias, in this case primarily due to the higher likeliness of patients with more advanced disease to undergo SL. Secondarily, selection algorithms for SL were not available and likely varied greatly per institution, which could skew results as well as introduce a considerable amount of selection bias when the surgeon decides on which patients apply for SL. In addition, details on periprocedural aspects of SL were not described in detail for each patient. Therefore, the thoroughness of SL and whether or not liver ultrasonography was performed could not be verified. Furthermore, the median time between primary surgery and re-resection in iGBC patients was 75 days, whereas 4 to 8 weeks have been suggested in literature as the optimal time interval to re-resection [21]. Thus, the chance of recurrence was higher in a part of the iGBC group due to this delay. Finally, for some patients, the exact location of DD was not clearly reported. For example, no differentiation was made between surface or deep parenchymal liver metastasis, and it was therefore impossible to record whether SL could have detected the metastasis.

In conclusion, SL before planned resection for pGBC obviates a nontherapeutic laparotomy in one in five patients. In iGBC patients, SL has a lower yield but is indicated after primary resection for cholecystitis and after initial R1/R2 resection due to their highest risk of DD.

## Data Availability

The datasets generated during and/or analysed during the current study are available from the corresponding author on reasonable request.
